# Staphylococcus aureus associated with surgical site infections in Western Kenya reveals genomic hotspots for pathogen evolution

**DOI:** 10.1099/acmi.0.000734.v4

**Published:** 2024-06-27

**Authors:** Nyabera Nicholas Mogoi, Anthony Wawire Sifuna, Patrick Kirsteen Okoth, Oleg Reva, Rose Malaba, Ruth Negesa, Kuloba Peter Nyongesa, Kombo Ezra Osoro, Martin Welch

**Affiliations:** 1 Department of Biological sciences, Masinde Muliro University of Science and Technology, Kakamega, Kenya; 2 Department of Medical Biochemistry, Masinde Muliro University of Science and Technology, Kakamega, Kenya; 3 Department of Biochemistry, Genetics and Microbiology, Centre For Bioinformatics And Computational Biology, University of Pretoria, Pretoria, South Africa; 4 Kakamega County General Teaching and Referral Hospital, Kakamega, Kenya; 5 Department of Medical Microbiology and Parasitology, Masinde Muliro University of Science and Technology, Kakamega, Kenya; 6 Department of Biochemistry, University of Cambridge, Cambridge, UK

**Keywords:** antimicrobial resistance, genomic islands, nosocomial infection, plasmids, *Staphylococcus aureus*, sub-Saharan Africa, virulence

## Abstract

**Objectives.***Staphylococcus aureus* is one of the most common pathogens attributed to hospital infections. Although *S. aureus* infections have been well studied in developed countries, far less is known about the biology of the pathogen in sub-Saharan Africa.

**Methods.** Here, we report on the isolation, antibiotic resistance profiling, whole genome sequencing, and genome comparison of six multi-drug resistant isolates of *S. aureus* obtained from a referral hospital in Kakamega, Western Kenya.

**Results.** Five of the six isolates contained a 20.7 kb circular plasmid carrying *blaZ* (associated with resistance to β-lactam antibiotics). These five strains all belonged to the same sequence type, ST152. Despite the similarity of the plasmid in these isolates, whole genome sequencing revealed that the strains differed, depending on whether they were associated with hospital-acquired or community-acquired infections.

**Conclusion.** The intriguing finding is that the hospital-acquired and the community-acquired isolates of *S. aureus* belonging to the same genotype, ST152, formed two separate sub-clusters in the phylogenetic tree and differed by the repertoire of accessory virulence genes. These data suggest ongoing adaptive evolution and significant genomic plasticity.

## Data Summary

The disproportionate low presentation of whole genome sequencing (WGS) data and genome comparison results in sub-Saharan Africa (SSA), coupled with restricted diagnostic capabilities continues to limit AMR surveillance data on hospital acquired *Staphylococcus aureus* infections and management. The study points to a likelihood of well-established and circulating *S. aureus* sub-type in major health facilities in sub-Saharan Africa. Sequence reads for Chromosomes and plasmids in this study are available in the NCBI under accession numbers CP121234, CP121235, CP118688, CP118689, CP121238, CP121239, CP121240, CP121241, CP121242, CP121243, CP121244, CP121236, and CP121237. Supplementary files are available with online version of this article.

## Introduction

*Staphylococcus aureus* has emerged as a clinically important pathogen, often characterized by a high level of antimicrobial resistance (AMR) [1]. In 2019 alone, *S. aureus* was ranked as a leading cause of death, accounting for more than 1.1 million mortalities globally [2]. Moreover, reports have indicated an unprecedented resurgence of antibiotic resistance in the last decade [3,4], posing challenges to healthcare systems worldwide [5,7]. Globally, a number of prominent circulating clones of *S. aureus* have been reported, including the important global clones ST8 (CC8) [8,10], CC5 [11], CC1, CC22, CC30, CC45 and ST239 (CC239) [12,13]. However, based on the rather small number of genomic analyses carried out to date, fewer *Staphylococcal* subtypes/sequence types (STs) have been reported for sub-Saharan isolates. Nevertheless, ST8, ST5, and ST239 [14] have all previously been associated with hospital and community infections in the region [15,16], and ST152 and ST121 have been associated with community-acquired infections [16,20]. Indeed, the prevalence of ST152 has been reported to be high in hospital settings in Kenya [10,21, 22], indicating that this clone may be a regionally important. Another regionally-important ST may be ST88, which has been isolated from several skin and soft tissue infections, including surgical site infections [23]. These limited studies notwithstanding, surveillance and monitoring of important pathogens generally, and *S. aureus* specifically, associated with multi-drug resistance in the region remains extremely limited. This is a problem because *S. aureus* remains a common cause of wound infections, pneumonia, urinary tract infections, and bacteraemia, and consequently, represents a major infectious disease burden for hospitals in the region [16,24].

In the current work, we analyse a collection of *S. aureus* recovered from both hospital-acquired infections and community-acquired infections at a referral hospital in Kakamega, Western Kenya. This facility has an established antimicrobial stewardship (AMS) programme, but like in many similar facilities in sub-Saharan Africa, the programme is poorly funded and does not function optimally. Consequently, pathogens such as *S. aureus* remain a significant healthcare/infection problem [25]. Surgery is one of the many risk factors for infection with *S. aureus* [26]. The occurrence and transmission of surgical site infections (SSIs) is therefore of concern, even for patients undergoing minor operations. The problem is further exacerbated by the rise in bacterial pathogens that are resistant to antimicrobial agents [27] and by the high incidence of immuno-suppressive HIV in the region. Collectively, these factors contribute to an escalating burden of hospital infections in Kenya [13,28], yet we have only limited information about how these infections are transmitted in the hospital setting. In the current study, we move towards addressing these issues by (*i*) isolating a selection of *S. aureus* from surgical wound infections, (*ii*) characterizing the antimicrobial resistance profile of these isolates, (*iii*) carrying out whole genome sequence analysis in a selection of the isolates. Our data reveal possible drivers behind evolutionary segregation in closely-related lineages of *S. aureus*.

## Methods

### Study design

This cross-sectional study was carried out between March 2021 and February 2022 at the Kakamega County General Teaching and Referral Hospital (KCGTRH); the largest referral hospital in Western Kenya. Patients with surgical site infections (SSIs) from general surgery and post-natal wards were enrolled after consenting to participate in the study. Clinical and demographic data were collected for each participant using a questionnaire and from previous on-file information. These data included the age, weight and sex of each patient, ward and type of operation, SSI and extent of infection, length of hospital stay(s) and antibiotic treatment history. We defined hospital-acquired isolates as being from patients requiring admission for caregiving reasons, whereas community-acquired isolates were from patients treated at the facility but who did not require admission. In this study, the cutoff value for HAIs was surgical wound infection occurring up to 3 days (72 h) after discharge, or up to 30 days after a surgical procedure. These were infections not present at the time of admission to hospital. On the other hand, infections present or incubating at the time of hospital admission or that develop within the first 48 h of admission were considered CAIs. In some cases, CAIs also referred to infections that were acquired outside of healthcare facilities in the community setting. The following designations were used to categorize the associated isolates: ID – isolate likely acquired in the hospital from general surgical ward(s), OD – isolate likely acquired in the community by patients that attending a general surgical ward, and IB – isolate likely acquired in the hospital from post-natal wards. Wounds were assessed and classified as described by Herman & Bordoni (2022) [29] as class I-IV. The identification for recruitment of 70 patients was based on clinical records with the help of clinicians. After consenting to the study, patients presenting with post-surgical infections occurring up to 4 weeks after minor or major operation, fell under inclusion criteria. Patients with community acquired infections like open fractures and furuncles were considered in the exclusion criteria. Sample collection was done by certified clinicians. Samples were collected by swabbing the SSI using two sterile cotton swabs. The swabs were placed in Amies transport media [30] and processed at the facility microbiology laboratory within 2 h of collection. The first swab was used for Gram-staining, and the second swab was used to recover *Staphylococci* on blood agar (BA) and mannitol salt agar (MSA) plates. *Staphylococci*-positive colonies were confirmed using catalase and coagulase tests. Twenty-four isolates were recovered in all. For long-term storage, the isolates were suspended in trypticase soy broth supplemented with 15 % v/v glycerol and frozen at –80 °C.

### Antimicrobial sensitivity tests

Each of the 24 recovered *S. aureus* isolates was subjected to antibiotic susceptibility testing (Kirby Bauer disc diffusion technique). The antibiotic discs tested were ampicillin (AMP), amoxicillin (AMX), ceftazidime (CAZ), cefalexin (CEF), cephalothin (CEP), cephazolin (CFZ), clindamycin (CLM), ceftriaxone (CRO), cefuroxime (CXM), erythromycin (ERY)**,** gentamicin (GEN), oxacillin (OXA), penicillin (PEN), tetracycline (TET), and trimethoprim (TMP). The reference strain was *S. aureus* ATCC 29213. Relative susceptibility (resistant, intermediate, susceptible) to each antibiotic was assessed based on CSLI guidelines.

### DNA isolation, sequencing and assembly

DNA was obtained from selected isolates using the ZymoGen Genomic DNA Extraction Kit (17 062 Murphy Ave., Irvine, CA 92614) following the manufacturer’s instructions. The isolated gDNA was quantified using a NanoDrop 2000c spectrophotometer (Thermo Scientific, USA), and DNA integrity was assessed by agarose (1 % w/v) gel electrophoresis. Subsequently, the gDNA was sheared into short fragments, end-repaired, A-tailed, and ligated to Illumina adapters. Library quantification was performed using Qubit, and library size distribution was assessed using a Bioanalyzer. Paired-end sequencing was conducted on an Illumina HiSeq 2500 platform (Novogene Co., Ltd). Raw, paired-end reads were trimmed using a pipeline implemented in Unipro UGENE v.44.0 [31], based on CASAVA 1.8 with default settings for adapter removal, read trimming, and quality filtering. The quality Q-score threshold for trimming and filtering was set at 20. For genome assembly, a combinatory approach of *de novo* assembly using SPAdes v3.15.3 [32] and DNA read mapping against a reference sequence by Bowtie2 [33] was used. *S. aureus* ATCC BAA-39 [CP033505.1] was selected as the reference genome as a representative of multidrug resistant organism with multiple inserts of virulence and antibiotic resistance gene including the SCCmec cassette specific for the methicillin resistant MRSA clonal line [34]. Samtools 1.13 was utilized for sorting the resulting alignments of the trimmed DNA reads against the reference genome. Then, the default consensus sequence algorithm with an automatic gap removal implemented in Unipro UGENE v.44.0 was used to produce assembled sequences from the sorted alignments. In parallel, *de novo* assembled contigs were ordered against the reference sequence using the function Move Contigs function of Mauve v.20150226 [35]. Contigs, which were not aligned against the reference sequence, were identified as plasmid contigs using the mlplasmids 1.0.0 [36] tool and the mob_recon function from the Mob-Suite v3.1.7 utility (https://github.com/phac-nml/mob-suite) [37] with the default parameter setting. Ordered contigs were concatenated into a single sequence. The function ProgressiveMauve of the Mauve v.20150226 with match seed weight parameter set to 15 was used to align the sequence of concatenated contigs (termed subject sequence) against the consensus sequence obtained by read mapping (termed reference sequence). Identified gaps in the subject sequence were stored in a separate file using function Export Gaps of the Mauve v.20150226. An in-house programme GapClosure [38] was written on Python three to patch the gaps of the subject sequence by respective sequences of the reference sequence. Programme GapClosure ignored gaps shorter than 10 bp. Subsequently, the original reads were mapped against the assembled sequences to verify the patches followed by obtaining new consensus sequences. Possible duplications of contigs in the assembled genomes were identified by the nucmer, rclT, and show-coords functions of MUMmer 4.0.0rc1 [39]. Quality of genome assembly was controlled by CheckM v.1.0.18 [40] provided at the KBase web-portal (https://www.kbase.us/). The consensus sequences were annotated using RAST server (https://rast.nmpdr.org/; [41]) with the automatic error-fixing option enabled. For genome annotation, *Staphylococcus aureus* (taxid: 1280) was set as the target bacterial genus with genetic code 11. Other parameters of the RAST annotation robot were set by default. All assemblies were rearranged using BioPython v1.81 to start at the position of 400 bp upstream of the *dnaA* chromosomal replication initiator in leading strands, corresponding to the chromosomal replication origins. The resulting chromosomal and plasmid sequences were submitted to the NCBI repository in FASTA format. They were automatically annotated by the NCBI Prokaryotic Genome Annotation Pipeline (PGAP-6.6) [42]. Accession numbers of the genomes, CheckM assembly quality parameters and other supporting information are shown in Table 1.

**Table 1.** Sequence annotation summary shows chromosome and plasmid annotation of predicted genes versus the actual number of protein-coding genes

Isolate ID	Replicon	Length (bp)	CDS	NCBI accession	CheckM parameters	
					Completeness	Contamination
OD001	Chromosome	2 479 797	2 809	CP121234	95 %	0.04 %
	Plasmid	20 767	23	CP121235	na	na
ID003	Chromosome	2 525 702	2 608	CP118688	98 %	0.08 %
	Plasmid	20 807	23	CP118689	na	na
IB011	Chromosome	2 398 889	2 439	CP121238	97 %	0 %
	Plasmid	20 785	23	CP121239	na	na
OD028	Chromosome	2 473 420	2 632	CP121240	95 %	0.08 %
	Plasmid	20 808	24	CP121241	na	na
ID029	Chromosome	2 652 936	2 860	CP121242	96 %	0.12 %
	Plasmid_1	14 849	19	CP121243	na	na
	Plasmid_2	10 162	13	CP121244	na	na
IB010	Chromosome	2 490 038	2 540	CP121236	98 %	0.99 %
	Plasmid	20 814	23	CP121237	na	na

### Genome analyses

Antibiotic resistance genes were identified using a local installation of the RGI v.6.0.3 software [43]. Virulence-associated genes were screened through the Virulence Factor Database (VFDB) [44] using a local installation of Abricate v.1.0.1. Both software tools, Abricate and RGI, were run with default parameter settings. Specifically, Abricate was configured for a minimum coverage of 80 % and minimum identity of 90 %. Assembled genome sequences in FASTA format were used as inputs for the programmes Abricate and RGI. To ensure that the strain-specific appearance of various virulence and drug resistance factors were not attributed to errors in genome assembly, the genomic sequences of all isolates were aligned using Mauve v.20150226 against the longest chromosomal sequence of strain ID003. Corrections were made to these genomes using the GapClosure tool, as explained in the Methods section. Further verification processes involved mapping the original DNA reads back to the amended sequences. The resulting consensus sequences were then re-annotated using the RAST Server.

To determine sequence types (STs), the FASTA chromosomal sequence files were uploaded to PubMLST at https://pubmlst.org/organisms/staphylococcus-aureus [45]. In the sequenced genomes, clusters of orthologous genes (COGs) were predicted using the programme OrthoFinder with default parameters [46]. The alignment of each COG’s sequences was performed using muscle [47]. Gblocks [48] was used to remove ambiguous parts of the alignments. Subsequently, BioPython v1.81 scripts were employed to concatenate the COG alignments into a superstring alignment. This superstring alignment was utilized for further phylogenetic inferences using a Neighbour-Joining algorithm, implemented in Neighbour (part of the phylip package 3.69) (https://evolution.genetics.washington.edu/phylip.html), with *S. aureus* ATCC BAA-39 [CP033505], *S. epidermidis* ATCC 12228 [NC_004461], and *S. saprophyticus* ATCC 15305 [NC_007350] serving as references for out-grouping. Sequence identity of plasmids was estimated by aligning their nucleotide sequences using the ClustalW algorithm implemented in the programme BioEdit v7.2.5 [49], which produced a sequence identity table for the created alignment. The SeqWord Gene Island Sniffer was employed to identify horizontally-acquired Genomic Islands (GI) [50,51]. Gene annotation of predicted GIs was checked individually to exclude false predictions of genomic regions containing housekeeping genes with alternative k-mer patterns, such as clusters of rRNA, genes for ribosomal RNA and giant genes with multiple repeats. All the programmes mentioned above were run with default parameter settings.

## Results

We wanted to understand the biology and epidemiology of *S. aureus* in a referral hospital in Western Kenya. We began by recovering 24 *S*. *aureus* isolates from SSIs to determine their antibiotic susceptibility profile.

### Antibiotic susceptibility profiles of *S. aureus* isolates

The AMR profile of each of the 24 *S*. *aureus* isolates is shown in Table S1, available in the online version of this article. At the time the isolates were collected, the hospital was using a third-generation cephalosporin, ceftriaxone (CRO) and a second-generation β-lactam, oxacillin (OXA) as the main prophylactic drug of choice for patients undergoing surgery. Of the 24 isolates tested, six were consistently resistant against multiple antibiotics, including CRO and OXA. These six isolates were designated ID003 and ID029 (both acquired in the hospital surgical unit), IB011 and IB010 (both acquired in the hospital post-natal ward), and OD028 and OD001 (both likely community-acquired). Six strains showing a broad spectrum of antibiotic resistance were selected for whole genome sequencing as representatives of hospital- and community-acquired *S. aureus* isolates.

#### Genotyping and phylogenetic relatedness of the isolates

**Fig. 1.**
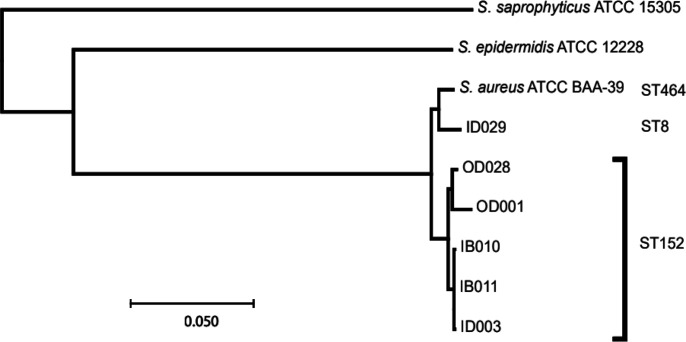
Phylogenetic tree of *S. aureus* isolates (cf. three ATCC reference strains) with MLST of each isolate indicated. Note the sub-culstering of the two community-acquired isolates (OD028 and OD001) and the three hospital-acquired isolates (ID003, IB011 and IB010) within ST152. This tree were based on amino acid sequences (concatenated alignments of all homologous proteins). Scale bar represents evolutionary distances between aligned protein sequences calculated using the JTT substitution model.

Whole genome sequences of six selected isolates were obtained using Illumina MiSeq sequencing and assembly, as described in the Methods section. The quality of the assemblies was evaluated using CheckM v.1.0.18 software, which revealed genome completeness ranging from 95 % (OD028) to 98 % (IB010 and ID029), and a contamination level of approximately 0.08 % for all assemblies. The isolates were assigned to sequence types following a PubMLST analysis. ID029 belongs to ST8, whereas all other selected isolates belong to ST152, including the hospital-acquired (ID003, IB011, IB010) and community-acquired (OD001, OD028) strains. To obtain a finer-grained analysis of the relationships between the isolates, we first identified the core gene set common to all six isolates and to three reference genomes: *S. aureus* ATCC BAA-39 [CP033505], *S. epidermidis* ATCC 12228 [NC_004461] and *S. saprophyticus* ATCC 15305 [NC_007350]. This yielded a collection of 1 839 clusters of orthologous genes (COGs). For each isolate, these genes were then translated into corresponding amino acid sequences, aligned separately, and the alignments were concatenated to yield an assembly comprising 475 577 amino acid residues. The concatenated amino acid sequence alignment was used to infer a phylogenetic tree using the Neighbour-Joining algorithm (Fig. 1). The phylogenetic tree was congruent with the MLST predictions, and further, allowed us to discriminate the hospital-acquired and community-acquired isolates, which formed two distinguishable sub-clusters within ST152.

Genomes of all selected *S. aureus* isolates comprise plasmids (Table 1). The plasmid sequence alignment is shown in Fig. S1. The most immediately obvious feature is that the plasmids of all ST152 isolates showed 99 % identity of their nucleotide sequences. These homologous plasmids encode 23 genes, including pairs of recombinases and transposases. The transposases flank a cassette of six genes comprising class A β-lactamase (*blaZ*), β-lactam sensor/signal transducer (*blaR1*), possible penicillinase repressor (*blaI*), and three genes of unknown function. blastn search through the NCBI nr database revealed an unnamed plasmid from *S. aureus* strain 13 420 [CP021142], and plasmid pLDNT_611 from *S. aureus* strain NT_611 [CP080252] as the top hits. In several other published genomes of *S. aureus*, similar plasmid sequences were assigned as being chromosomal. Notably, all of the *S. aureus* strains from NCBI containing these plasmid sequences belonged to ST152.

Two smaller plasmids were found in the ST8 isolate, ID029. The longer of the two shared several homologous segments with the ST152-associated plasmid (Fig. S1). However, instead of a *blaZ*-containing cassette, it contained four *staphylococcal* enterotoxin-encoding genes. The smaller ID029 plasmid carried an efflux transporter *qacA* involved in antiseptic resistance.

### Virulence determinants

**Fig. 2.**
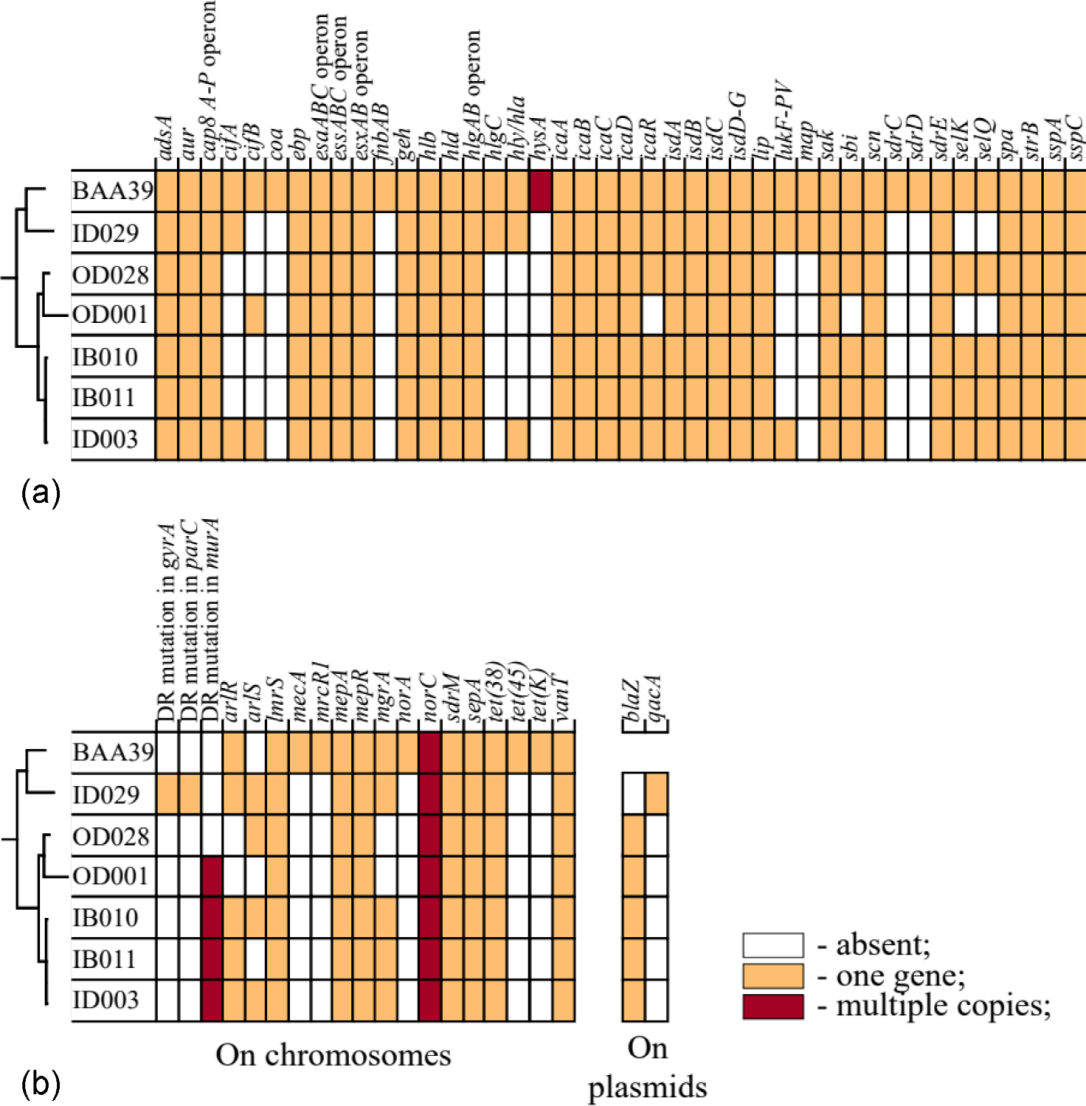
Distribution of virulence and drug resistance genes in the genomes of the selected *S. aureus* isolates and the reference strain *S. aureus* ATCC BAA-39. (**a**) Virulence genes identified by searching through the VFDB database using the programme Abricate. (**b**) Antibiotic resistance genes and mutations identified by searching through the CARD database using the programme RGI.

Virulence genetic determinants were searched in the VFDB database using the programme Abricate. The previously sequenced strain *S. aureus* ATCC BAA-39 was used as a reference [34]. The analysis identified multiple virulence-associated genes, as summarized in Fig. 2a. The highest number of virulence genes was found in the reference MRSA strain *S. aureus* BAA-39, followed by the hospital-acquired isolates ID029 and ID003, which belong to different MLST types. Unlike other ST152 isolates, strain ID003 contains the *cifAB* fibrinogen-binding proteins, the *hly/hla* alpha-hemolysin precursors, and the *lukF* Panton-Valentine leukocidin precursor, which were also found in ID029. Additionally, ID029 possesses the *hlgC* gamma-hemolysin component, absent in the other isolates. The community-acquired ST152 isolates, OD001 and OD028, have a smaller number of virulence-associated genes. OD001 lacks the *icaR* transcriptional regulator of intercellular adhesion, the IgG-binding protein *sbi*, and the staphylococcal enterotoxins K and G, encoded by *selK* and *selG*, respectively. The latter two genes were also absent in the genome of ID029, but the other isolates possessed all these genes.

Due to the stringent gene search parameters set by default in the programme Abricate, several putative virulence associated genes were overlooked by this search. Specifically, while the programme failed to identify virulence genes on the plasmids, an inspection of annotated genes revealed the presence of a cluster of staphylococcal enterotoxins R, J, T, and S in ID029’s larger plasmid. Additionally, ID029’s shorter plasmid harbours *qacA* multidrug efflux pump and its transcriptional regulator *qacR*. This efflux pump is a component of the cell’s defence mechanism against toxic compounds, including many antimicrobial agents [52,53].

### Antibiotic resistance genetic determinants

Resistance to antibiotics can be conferred by mutations in target proteins or by the acquisition of drug resistance genes, such as efflux pumps, alternative versions of the target proteins, or enzymes that degrade antimicrobial compounds.

Several mutations associated with acquired antibiotic resistance, as reported in the literature, were discovered in the selected strains using the programme RGI (Fig. 2b). All strains belonging to ST152 have two mutations in MurA transferase, E291D and T396N. Both of these mutations have been observed in the context of fosfomycin resistance in *S. aureus* [54]. Fosfomycin targets the MurA enzyme in bacteria, inhibiting cell wall synthesis by blocking the formation of N-acetylmuramic acid, a critical component of the bacterial cell wall. Mutations in the MurA transferase gene alter the structure of the MurA enzyme, decreasing the binding affinity of fosfomycin. This structural change prevents fosfomycin from effectively inhibiting the enzyme, leading to resistance.

*S. aureus* ID029 (ST8) has two other mutations in GyrA DNA gyrase subunit A (S84L) and ParC DNA topoisomerase IV subunit A (S80Y). These two mutations reduce the binding affinity of fluoroquinolones to these enzymes, diminishing their inhibitory effectiveness and thereby conferring resistance [55,56].

Several genes associated with antimicrobial resistance are encoded on the chromosomes of each isolate (Fig. 2b). Most of these genes, which encode broad-spectrum efflux pumps, were found in all isolates, whereas *arlSR* and *mgrA* were absent or incomplete in the community isolates OD001 and OD028. In contrast, the reference strain *S. aureus* ATCC BAA-39 contains additional efflux pumps, *tet(45*) and *tet(K*), as well as the SCCmec methicillin resistance cassette with genes *mecA* and *mrcR1*. These genes were absent in the genomes of the selected strains.

### Horizontally transferred genetic islands

**Fig. 3.**
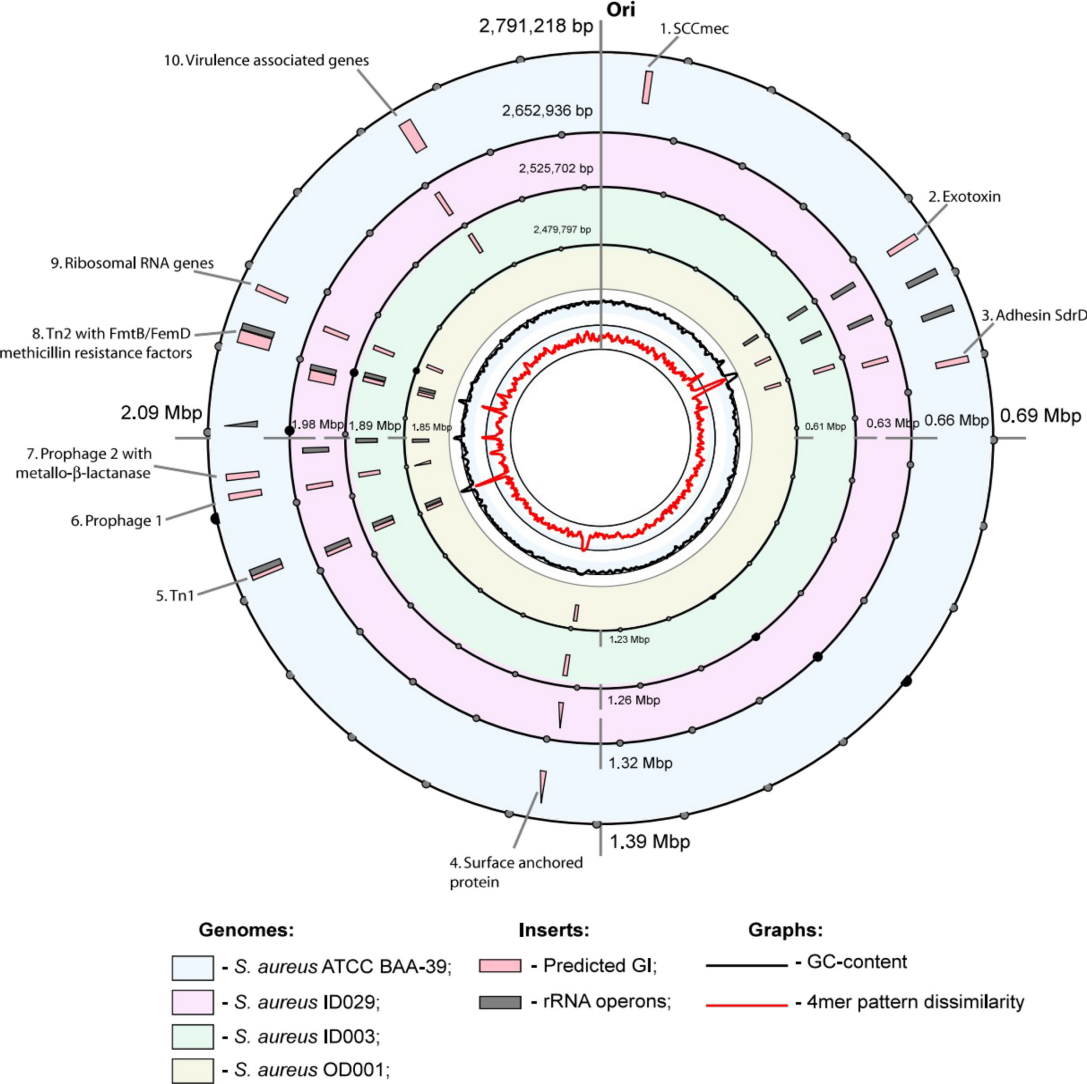
Distribution of genetic islands (GIs) on the chromosomes of *S. aureus*. The outermost ring represents the chromosome of the reference strain *S. aureus* ATCC BAA-39, which is followed by graphical representations of the chromosomes of the isolates ID029 (represents ST8 genotype); ID003 (represents hospital isolates of ST152); and OD001 (represents community isolates of ST152). All chromosomes were aligned to start from putative replication origin (Ori) locations, which were selected 400 bp upstream of the genes for chromosomal replication initiator protein DnaA. Locations of inserts of GIs and rRNA clusters are depicted respectively by pink and dark grey boxes. Fluctuations of GC-content and 4-mer pattern dissimilarities in five kbp sliding windows are shown by black and red-coloured histograms, respectively.

Horizontally-acquired genetic islands (GIs) were identified using the SeqWord Sniffer programme, which was designed to identify relatively large integrons and prophages. It measures differences in k-mer patterns of local sliding windows compared to the overall k-mer pattern of the chromosome [50]. The predicted locations of GIs in the chromosomes of the selected *S. aureus* isolates are shown in Fig. S2. GIs locations and lists of genes found within GIs are shown in Table S1. The distribution of GIs in the selected isolates was then compared with that of the reference strain *S. aureus* ATCC BAA-39 (Fig. 3). It was found that the locations of horizontally acquired inserts in chromosomes of *S. aureus* are quite conserved. The regions in Fig. 3 are numbered from 1 to 10, following the order of the inserts in the reference genome, where the number of inserts is the largest.

Insert #1, located near the replication origin in the reference strain, corresponds to the SCCmec methicillin resistance cassette. This insert was specific to MRSA strains, as was Insert #2, which contains a gene encoding an exotoxin. GI #3 appears to be a historical insert containing the *sdrD* and *sdrE* adhesins. It is present in all *S. aureus* strains; however, the programme did not identify it as a foreign insert in strain IB010, due to the similarity of its 4-mer pattern to the surrounding chromosomal loci. Region #4 encompasses genes for giant staphylococcal surface-anchored proteins, characterized by an alternative frequency of 4-mers. Therefore, this region should be considered a false-positive prediction.

GIs #5 and #8, identified as transposable elements Tn1 and Tn2 in Fig. 3, are associated with clusters of tRNA genes upstream of rRNA clusters. The terminal genes of these inserts are identical, consisting of a membrane-associated PTS transporter subunit and the transposase IS1181 annotated as the ‘mobile element protein’. Trails of integrated genes are found on the chromosome upstream of the transposase. In Tn1, these genes are conserved, including two *glnQ*-like ABS transporters and the *queG* epoxyqueuosine reductase. In contrast, Tn2 remains active, capable of integrating additional horizontally transferred gene cassettes, as evidenced by the larger size of these regions in the reference genome and in ID029. The genomes of all strains examined in this study contain the *fmtB*/*mrp* cell wall biosynthesis and *femD* phosphoglucosamine mutase genes involved in methicillin resistance [56]. These genes are located at the 5′-terminal part of the inserts, opposite the transposases. Upstream of the transposase, an arginase, a bacterial checkpoint controller *disA*, and the diadenylate cyclase *spyDAC* are found. The presence of intermediate genes varies across different strains. ID029, for example, harbours several additional copies of *fmtB*/*mrp*-like genes and an uncharacterized secreted protein was found in the reference strain.

GI #6 is identified as a historical prophage with remnants of phage-associated genes. Nearby, the reference strain also harbours an additional prophage, GI #7, which includes a metallo-beta-lactamase superfamily domain protein alongside phage genes. This insert was not found in other *S. aureus* strains sequenced in this study. Region #9 represents another false positive prediction common to all *S. aureus* strains, featuring a cluster of ribosomal protein genes. This DNA locus is distinguished by an alternative 4-mer nucleotide usage pattern.

GI #10 likely serves as an active chromosomal hotspot that facilitates new genetic acquisitions through horizontal gene transfer. Both the reference strain BAA-39 and ID029 contain the *sasG* virulence-associated cell-wall-anchored protein here, which plays a role in binding to the squamous nasal epithelium [57], followed by several transcriptional regulators, enzymes involved in the UTP-glucose-1-phosphate transformation, and various fibronectin-binding proteins. ID003 is the sole isolate within the ST152 group featuring an insertion at this locus, which includes multiple genes for fibronectin-binding proteins and one UTP-glucose-1-phosphate uridylyltransferase gene. Significantly, the strain ID003 from the ST152 group shares this characteristic with ID029. All the virulence genes identified within the GIs were not detected by the Abricate search against the VFDB database, conducted with the default stringent identity and coverage parameters (Fig. 1).

## Discussion

In this study, we compared the genomes of hospital-acquired and community-acquired isolates of multi-drug resistant *S. aureus*. Five out of the six selected strains were classified as ST152, encompassing both hospital-acquired and community-acquired isolates. A single hospital-acquired isolate, ID029, was identified as ST8. These findings collectively suggest that hospitals in this region may harbour distinct *S. aureus* sequence types.

The observed high level of β-lactam resistance among the ST152 isolates is likely due to the *blaZ*-carrying plasmid. Resistance to fosfomycin was noted to arise from mutations in the MurA transferase. The ST8 isolate, ID029, exhibited extensive antibiotic resistance, primarily through mutations in the target proteins GyrA and ParC. Nonetheless, the resistance mechanisms identified do not fully account for the wide range of antibiotic resistance observed in our examined isolates, indicating the potential involvement of additional antibiotic resistance mechanisms [58,59].

A notable finding was the distinction between the two community-acquired ST152 isolates and their hospital-acquired counterparts, with the former group having fewer horizontally acquired GIs, virulence, and antibiotic resistance genes. This highlights the significant genome plasticity of *S. aureus*, enabling adaptive evolutionary changes through gene exchange and horizontal gene transfer.

## Conclusion

Here, we demonstrate that *S. aureus* ST152 is locally abundant among clinical isolates with a multi-drug-resistant phenotype in Kakamega, Western Kenya. All ST152 isolates carried similar plasmids encoding several antibiotic resistance genes, including the *blaZ* β-lactamase. Phylogenetic analysis and genome comparison suggest ongoing evolution among the ST152 isolates, leading to the segregation of hospital-acquired and community-acquired lineages. An inspection of PubMLST database records revealed that out of 121 recorded ST152 isolates, 98 strains (81 %) were isolated in African countries. Another multi-drug-resistant isolate, ID029, possessing two virulence plasmids, belongs to ST8. Pathogens of this genotype are common among hospital *S. aureus* isolates; however, according to 3 726 records in the PubMLST database, the ST8 genotype is dominant in the USA and Europe but rare in Africa. This observed geographic separation of *S. aureus* sequence types warrants further study to determine whether it is associated with adaptation to specific human populations or antibiotic treatment protocols in different regions. Another plausible explanation is that these rapidly evolving lineages have emerged recently and are dominant in the regions where they appeared.

## supplementary material

10.1099/acmi.0.000734.v4 Uncited Supplementary Material 1.

## Abbreviations

AMR: antimicrobial resistance
AMS: antimicrobial stewardship
Blaz: beta lactamsa Z
CAIs: community-acquired infections
CC: clonal complex
DNA: deoxyribonucleic acid
HAIs: hospital-acquired infections
HIV: human immunodeficiency virus
IB: inpatient, postnatal ward
ID: inpatient, general ward
KCGTRH: Kakamega County General Teaching and Referral Hospital
MRSA: methicilin resistant *Staphylococcus aureus*
OD: outpatient, general ward
RGI: resistance gene identifier
*S. aureus*: *Staphylococus aureus*
SSA: sub-Saharan Africa
SSIs: surgical site infections
ST: sequence type
WGS: whole genome sequencing
