# Supplementary material for: Staphylococcus aureus associated with surgical site infections in Western Kenya reveals genomic hotspots for pathogen evolution
Source: Access Microbiol. 2024 Jun 27;6(6):000734.v4. doi: 10.1099/acmi.0.000734.v4 (PMC11261728; doi:10.1099/acmi.0.000734.v4)
Supplement: Uncited Supplementary Material 1. [file acmi-6-00734-s001.pdf]

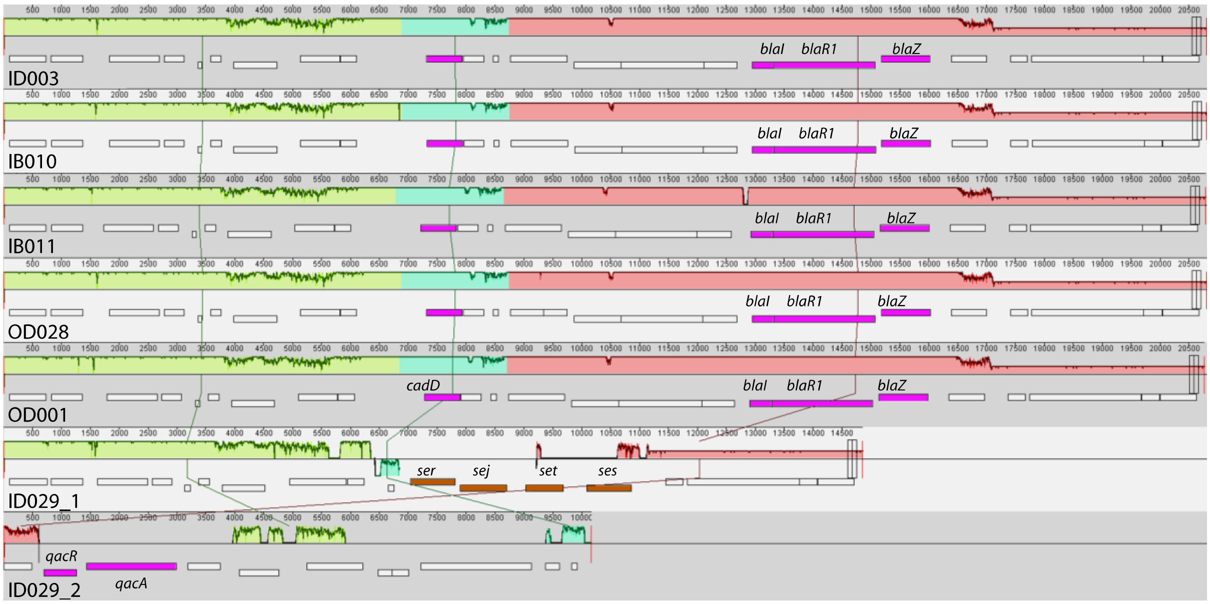

Supplementary Figure S1. Alignment of the plasmid sequences found in the *S. aureus* isolates (generated by Mauve). Note the clear similarity in terms of gene content and synteny of the ST152 group plasmids (ID003, IB010, IB011, OD028 and OD001). Pink color depicts antibiotic resistance genes; brown - virulence genes. Colored histograms show the level of DNA identity between plasmid sequences. Different colors of the histograms depict different Maximum Unique Match (MUM) regions identified in the plasmid sequence alignment.

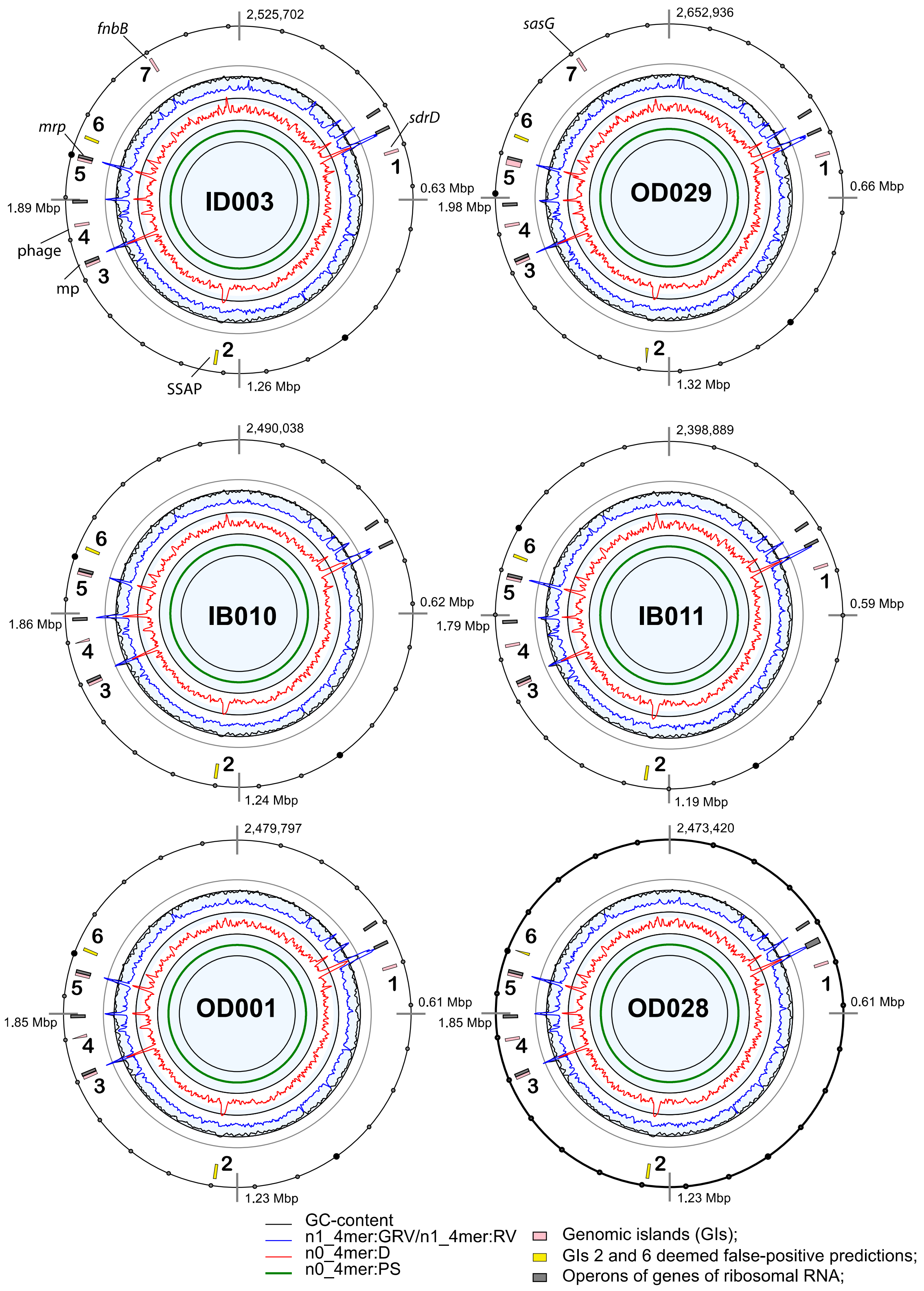

Supplementary Figure S2. Atlas maps of distribution of genetic islands (GIs) on the chromosomes of the selected *S. aureus* isolates.

Supplementary Table S1. Genes covered by genetic islands of *Staphylococcus aureus* isolates.

Strain	GI	Location	CDS
S. aureus **ATCC BAA-39 [CP033505]**			
	GI #1	54000..68099	MaoC domain protein, rev[54251..54680]
			Glycerophosphoryl diester phosphodiesterase, rev[54776..55520]
			Hydroxymethylglutaryl-CoA synthase, rev[56275..56698]
			Replication initiation protein, topoisomerase, rev[56714..57515]
			plasmid recombination enzyme type 3, rev[58156..59398]
			Tetracycline resistance, MFS efflux pump -> Tet(K), rev[59583..60963]
			hypothetical protein, dir[61202..61616]
			Organomercurial lyase, rev[61810..62461]
			Mercuric ion reductase, rev[62542..64186]
			hypothetical protein, rev[64243..64582]
			hypothetical protein, rev[64755..65397]
			hypothetical protein, rev[65426..65747]
			Transcriptional regulator, MerR family, rev[65763..66171]
			pyridine nucleotide-disulfide oxidoreductase, rev[66470..67817]
	GI #2	438000..452099	Exotoxin 15, dir[439967..440669]
			Hypothetical protein, dir[440690..442193]
			hypothetical protein, rev[442300..442609]
	GI #3	598000..608099	adhesin SdrD, dir[601018..604729]
	GI #5	1918100..1930099	Epoxyqueuosine reductase, rev[1918385..1919513]
			Glutamine ABC transporter, ATP-binding protein GlnQ, rev[1919664..1920393]
			ABC transporter/substrate-binding protein/ABC transporter,permease protein, rev[1920379..1921570]
			Mobile element protein, dir[1922115..1923435]
			Membrane protein, rev[1923595..1924657]
	GI #6	2018100..2030099	Phage lysin, N-acetylmuramoyl-L-alanine amidase, rev[2018430..2018727]
			Staphylokinase, rev[2019417..2019909]
			Phage lysin, N-acetylmuramoyl-L-alanine amidase, rev[2020099..2020855]
			Phage holin, rev[2020866..2021121]
			Phage protein, rev[2021651..2022026]
			Phage protein, rev[2022081..2022369]
			Structural protein, phage associated, rev[2022559..2026342]
			Phage protein, rev[2026357..2027848]
	GI #7	2042000..2050099	Phage protein, rev[2044050..2044257]
			Dimeric dUTPase, rev[2044293..2044830]
			Phage protein, rev[2044822..2045071]
			hypothetical protein, rev[2045063..2045348]
			hypothetical protein, rev[2045344..2045794]
			Phage protein, rev[2045790..2045985]
			hypothetical protein, rev[2045981..2046368]
			Phage protein, rev[2046381..2046624]
			Phage protein, rev[2046627..2046996]

			Phage Holliday junction resolvase, rev[2047008..2047413]<br>Phage protein, rev[2047421..2047640]<br>Phage protein, rev[2047646..2048540]<br>Single-stranded DNA-binding protein, phage associated, rev[2048569..2049040]<br>Metallo-beta-lactamase superfamily domain protein in prophage, rev[2049040..2049526]
	GI #8	2210000..2226099	FmtB (Mrp) protein involved in methicillin resistance and cell wall biosynthesis, rev[2211318..2216919]<br>Phosphoglucosamine mutase / FemD, factor involved in methicillin resistance, rev[2217196..2218552]<br>putative secreted protein, rev[2218578..2219511]<br>Diadenylate cyclase spyDAC, Bacterial checkpoint controller DisA with nucleotide-binding domain, rev[2219512..2220322]<br>Arginase, rev[2220510..2221419]<br>Mobile element protein, rev[2221570..2222890]
	GI #10	2540000..2556099	Virulence-associated cell-wall-anchored protein SasG, rev[2541229..2545345]<br>Transcriptional regulator SarT, rev[2545649..2546006]<br>Transcriptional regulator SarU (accessory regulator U), dir[2546329..2547073]<br>UTP--glucose-1-phosphate uridylyltransferase, rev[2547283..2547769]<br>Fibronectin binding protein FnbB, rev[2548328..2551193]<br>Fibronectin binding protein FnbA, rev[2551873..2554963]
**ID029**			
	GI #2	544000..552099	Adhesin of unknown specificity SdrC, dir[544008..545769]<br>Adhesin of unknown specificity SdrC, dir[545765..546677]<br>Adhesin of unknown specificity SdrD, dir[547064..548219]<br>Adhesin of unknown specificity SdrD, dir[548206..550165]<br>Adhesin of unknown specificity SdrE, similar to bone sialoprotein-binding protein Bbp, dir[550196..550682]
	GI #4	1814100..1826099	Glutamine ABC transporter, ATP-binding protein GlnQ, rev[1814802..1815531]<br>ABC transporter, substrate-binding protein (cluster 3, basic aa/glutamine/opines) / ABC transporter, permease protein (cluster 3, basic aa/glutamine/opines), rev[1815517..1816411]<br>ABC transporter, substrate-binding protein (cluster 3, basic aa/glutamine/opines) / ABC transporter, permease protein (cluster 3, basic aa/glutamine/opines), rev[1816512..1816710]<br>Mobile element protein, dir[1817241..1818561]<br>Membrane protein, rev[1818714..1819776]
	GI #5	1914000..1924099	Staphylokinase, rev[1914609..1915101]<br>Phage lysin, N-acetylmuramoyl-L-alanine amidase (EC 3.5.1.28), rev[1915291..1916047]<br>Phage holin, rev[1916058..1916313]<br>Phage protein, rev[1916524..1916659]<br>hypothetical protein, rev[1916693..1916831]<br>Structural protein, phage associated, rev[1916845..1919593]<br>Phage protein, rev[1919608..1920580]

		Phage tail length tape-measure protein T, rev[1920803..1921364]
		Phage protein, rev[1921466..1921670]
		Hypothetical protein, SAV0881 homolog [SA bacteriophages 11, Mu50B], rev[1921710..1921890]
		Hypothetical protein, SAV0877 homolog [SA bacteriophages 11, Mu50B], rev[1921886..1922093]
		Hypothetical protein, PVL orf52 homolog [SA bacteriophages 11, Mu50B], rev[1922089..1922392]
		Hypothetical protein, SAB1734c homolog [SA bacteriophages 11, Mu50B], rev[1922384..1922567]
		hypothetical protein, rev[1922670..1922811]
		Hypothetical protein, PVL orf51 homolog [SA bacteriophages 11, Mu50B], rev[1922824..1923067]
		Phage Holliday junction resolvase, rev[1923084..1923489]
		Phage protein, rev[1923497..1923635]
GI #6	2074000..2092099	FmtB (Mrp) protein involved in methicillin resistance and cell wall biosynthesis, rev[2075284..2077102]
		FmtB (Mrp) protein involved in methicillin resistance and cell wall biosynthesis, rev[2077104..2078829]
		FmtB (Mrp) protein involved in methicillin resistance and cell wall biosynthesis, rev[2078971..2080714]
		FmtB (Mrp) protein involved in methicillin resistance and cell wall biosynthesis, rev[2080809..2082036]
		FmtB (Mrp) protein involved in methicillin resistance and cell wall biosynthesis, rev[2082242..2082737]
		Phosphoglucosamine mutase (EC 5.4.2.10) @ FemD, factor involved in methicillin resistance, rev[2083013..2084369]
		Uncharacterized secreted protein associated with spyDAC, rev[2084395..2085328]
		Diadenylate cyclase spyDAC
		Bacterial checkpoint controller DisA with nucleotide-binding domain, rev[2085329..2086139]
		Arginase (EC 3.5.3.1), rev[2086327..2087236]
		Mobile element protein, rev[2087387..2088695]
GI #8	2404000..2414099	Virulence-associated cell-wall-anchored protein SasG (LPXTG motif), binding to squamous nasal epithelial cells, rev[2405270..2409386]
		Transcriptional regulator SarT (Staphylococcal accessory regulator T), rev[2409690..2410047]
		Transcriptional regulator SarU (Staphylococcal accessory regulator U), dir[2410370..2411114]
		UTP--glucose-1-phosphate uridylyltransferase (EC 2.7.7.9), rev[2411384..2411837]
		UTP--glucose-1-phosphate uridylyltransferase (EC 2.7.7.9), rev[2411944..2412250]
		Fibronectin binding protein FnbA, rev[2412429..2412897]
		Fibronectin binding protein FnbA, rev[2412906..2413035]
		Fibronectin binding protein FnbB, rev[2413264..2413774]
		Fibronectin binding protein FnbB, rev[2413880..2414072]
***S. aureus* ID003**		
GI #1	506000..516099	Deoxyguanosine kinase (EC 2.7.1.113), rev[506399..506912]
		tRNA-specific adenosine-34 deaminase (EC 3.5.4.33), dir[506978..507449]

		Hydrolase, HAD superfamily, dir[507595..508465]
		Predicted flavoprotein, dir[508485..509052]
		Transcription termination factor Rho, dir[509595..510111]
		Adhesin of unknown specificity SdrD, dir[510262..511402]
		Adhesin of unknown specificity SdrD, dir[511401..513558]
		Adhesin of unknown specificity SdrC, dir[513554..513971]
GI #3	1734100..1746099	tRNA (cytidine(34)-2'-O)-methyltransferase (EC 2.1.1.207), rev[1734780..1735239]
		Epoxyqueuosine reductase (EC 1.17.99.6) QueG, rev[1735243..1736371]
		Glutamine ABC transporter, ATP-binding protein GlnQ, rev[1736522..1737251]
		ABC transporter, substrate-binding protein (cluster 3, basic aa/glutamine/opines) / ABC transporter, permease protein (cluster 3, basic aa/glutamine/opines), rev[1737237..1738428]
		Membrane protein, rev[1739034..1740096]
GI #4	1828100..1838099	Phage lysin, N-acetylmuramoyl-L-alanine amidase (EC 3.5.1.28), rev[1828759..1829056]
		Staphylokinase, rev[1829746..1830238]
		Phage lysin, N-acetylmuramoyl-L-alanine amidase (EC 3.5.1.28), rev[1830428..1831184]
		Phage holin, rev[1831195..1831450]
		Phage protein, rev[1831661..1831796]
		Phage protein, rev[1831912..1832074]
		Phage minor structural protein (ACLAME 95), rev[1832192..1832378]
		Structural protein, phage associated, rev[1832559..1834950]
		Phage tail length tape-measure protein T, rev[1834965..1836738]
		Phage tail length tape-measure protein T, rev[1836754..1836958]
		Phage protein, rev[1836980..1837181]
		Hypothetical protein, SAV0881 homolog [SA bacteriophages 11, Mu50B], rev[1837240..1837390]
		Hypothetical protein, SAV0877 homolog [SA bacteriophages 11, Mu50B], rev[1837386..1837593]
GI #5	1990100..2000099	FmtB (Mrp) protein involved in methicillin resistance and cell wall biosynthesis, rev[1990260..1991229]
		FmtB (Mrp) protein involved in methicillin resistance and cell wall biosynthesis, rev[1991188..1991422]
		FmtB (Mrp) protein involved in methicillin resistance and cell wall biosynthesis, rev[1991429..1991903]
		FmtB (Mrp) protein involved in methicillin resistance and cell wall biosynthesis, rev[1991899..1993192]
		Phosphoglucosamine mutase (EC 5.4.2.10) @ FemD, factor involved in methicillin resistance, rev[1993469..1994825]
		Uncharacterized secreted protein associated with spyDAC, rev[1994851..1995784]
		Diadenylate cyclase spyDAC
		Bacterial checkpoint controller DisA with nucleotide-binding domain, rev[1995785..1996595]
		Arginase (EC 3.5.3.1), rev[1996783..1997692]
GI #7	2296000..2306099	hypothetical protein, dir[2297262..2297529]

		hypothetical protein, dir[2297672..2297981]
		UTP--glucose-1-phosphate uridylyltransferase (EC 2.7.7.9), rev[2298042..2298495]
		UTP--glucose-1-phosphate uridylyltransferase (EC 2.7.7.9), rev[2298602..2298908]
		Fibronectin binding protein FnbA, rev[2299087..2299297]
		Fibronectin binding protein FnbB, rev[2299278..2301243]
		Fibronectin binding protein FnbB, rev[2301245..2301743]
		FIG01108157: hypothetical protein, rev[2301774..2302026]
		Fibronectin binding protein FnbB, rev[2302423..2302633]
		hypothetical protein, dir[2302661..2302922]
		Fibronectin binding protein FnbA, rev[2303096..2303927]
		Fibronectin binding protein FnbA, rev[2303889..2304468]
		hypothetical protein, rev[2304602..2304803]
***S. aureus* IB010**		
GI #3	1696100..1708099	Hypothetical protein SAV1852, rev[1696799..1697195]
		Hypothetical protein SAV1853, rev[1697700..1697850]
		Hypothetical protein SAV1854, rev[1697874..1698474]
		tRNA (cytidine(34)-2'-O)-methyltransferase (EC 2.1.1.207), rev[1698521..1698953]
		Epoxyqueuosine reductase (EC 1.17.99.6) QueG, rev[1698957..1700085]
		Glutamine ABC transporter, ATP-binding protein GlnQ, rev[1700229..1700958]
		ABC transporter, substrate-binding protein (cluster 3, basic aa/glutamine/opines) / ABC transporter, permease protein (cluster 3, basic aa/glutamine/opines), rev[1700944..1702135]
		Membrane protein, rev[1702471..1703533]
GI #4	1796000..1802099	Phage tail length tape-measure protein T, rev[1796704..1798474]
		Phage tail length tape-measure protein T, rev[1798490..1798673]
		Phage protein, rev[1798696..1798897]
		Hypothetical protein, SAV0881 homolog [SA bacteriophages 11, Mu50B], rev[1798896..1799052]
		Hypothetical protein, SAV0877 homolog [SA bacteriophages 11, Mu50B], rev[1799048..1799255]
		Dimeric dUTPase (EC 3.6.1.23), rev[1799291..1799828]
		Hypothetical protein, SAB1734c homolog [SA bacteriophages 11, Mu50B], rev[1799820..1800033]
		Hypothetical protein, Lmo2313 homolog [Bacteriophage A118], rev[1800029..1800479]
		Hypothetical protein, PVL orf51 homolog [SA bacteriophages 11, Mu50B], rev[1800549..1800792]
		Hypothetical protein, PVL orf50 homolog [SA bacteriophages 11, Mu50B], rev[1800795..1801164]
		Phage Holliday junction resolvase, rev[1801176..1801581]
		Phage protein, rev[1801589..1801808]
GI #5	1962100..1974099	FmtB (Mrp) protein involved in methicillin resistance and cell wall biosynthesis, rev[1963506..1964454]
		FmtB (Mrp) protein involved in methicillin resistance and cell wall biosynthesis, rev[1964461..1964674]
		FmtB (Mrp) protein involved in methicillin resistance and cell wall biosynthesis, rev[1964946..1965948]

		FmtB (Mrp) protein involved in methicillin resistance and cell wall biosynthesis, rev[1966009..1966141]
		Phosphoglucosamine mutase (EC 5.4.2.10) @ FemD, factor involved in methicillin resistance, rev[1966335..1967691]
		Uncharacterized secreted protein associated with spyDAC, rev[1967717..1968650]
		Diadenylate cyclase spyDAC
		Bacterial checkpoint controller DisA with nucleotide-binding domain, rev[1968651..1969461]
		Arginase (EC 3.5.3.1), rev[1969649..1970558]
***S. aureus* IB011**		
GI #1	478000..488099	tRNA-specific adenosine-34 deaminase (EC 3.5.4.33), dir[478041..478512]
		Hydrolase, HAD superfamily, dir[478658..479519]
		Predicted flavoprotein, dir[479534..480101]
		Antiadhesin Pls, binding to squamous nasal epithelial cells, dir[480417..480870]
		Adhesin of unknown specificity SdrD, dir[480998..482144]
		Adhesin of unknown specificity SdrD, dir[482143..484045]
		Adhesin of unknown specificity SdrE, similar to bone sialoprotein-binding protein Bbp, dir[484349..487700]
GI #3	1636100..1646099	Hypothetical protein SAV1853, rev[1636543..1636693]
		Hypothetical protein SAV1854, rev[1636717..1637317]
		tRNA (cytidine(34)-2'-O)-methyltransferase (EC 2.1.1.207), rev[1637364..1637670]
		Epoxyqueuosine reductase (EC 1.17.99.6) QueG, rev[1637674..1638814]
		Glutamine ABC transporter, ATP-binding protein GlnQ, rev[1638910..1639639]
		ABC transporter, substrate-binding protein (cluster 3, basic aa/glutamine/opines) / ABC transporter, permease protein (cluster 3, basic aa/glutamine/opines), rev[1639625..1640900]
		Membrane protein, rev[1640985..1642047]
GI #4	1724100..1736099	Aspartate aminotransferase (EC 2.6.1.1), dir[1724114..1725383]
		Beta-hemolysin, dir[1725417..1725618]
		Phage protein, dir[1726547..1726739]
		Involved in expression of fibrinogen binding protein, phage associated, rev[1726791..1727142]
		Phage lysin, N-acetylmuramoyl-L-alanine amidase (EC 3.5.1.28), rev[1727594..1727891]
		Staphylokinase, rev[1728346..1728838]
		Phage lysin, N-acetylmuramoyl-L-alanine amidase (EC 3.5.1.28), rev[1729028..1729784]
		Phage holin, rev[1729795..1730050]
		Phage protein, rev[1730261..1730396]
		Structural protein, phage associated, rev[1730430..1733244]
		Phage protein, rev[1733259..1734429]
		Phage tail length tape-measure protein T, rev[1734419..1735010]
		Hypothetical protein, SAV0881 homolog [SA bacteriophages 11, Mu50B], rev[1735228..1735378]

			Hypothetical protein, SAV0877 homolog [SA bacteriophages 11, Mu50B], rev[1735374..1735581]
	GI #5	1882100..1892099	FmtB (Mrp) protein involved in methicillin resistance and cell wall biosynthesis, rev[1882643..1883243]
			FmtB (Mrp) protein involved in methicillin resistance and cell wall biosynthesis, rev[1883374..1884886]
			Phosphoglucosamine mutase (EC 5.4.2.10) @ FemD, factor involved in methicillin resistance, rev[1885108..1886464]
			Uncharacterized secreted protein associated with spyDAC, rev[1886490..1887423]
			Diadenylate cyclase spyDAC
			Bacterial checkpoint controller DisA with nucleotide-binding domain, rev[1887424..1888234]
			Arginase (EC 3.5.3.1), rev[1888422..1889331]
***S. aureus* OD001**			
	GI #2	500000..510099	tRNA-specific adenosine-34 deaminase (EC 3.5.4.33), dir[500269..500740]
			Hydrolase, HAD superfamily, dir[500886..501759]
			Predicted flavoprotein, dir[501777..502344]
			Adhesin of unknown specificity SdrD, dir[502726..503872]
			Adhesin of unknown specificity SdrD, dir[503855..505829]
			Adhesin of unknown specificity SdrD, dir[506176..506680]
			Adhesin of unknown specificity SdrE, similar to bone sialoprotein-binding protein Bbp, dir[507072..510099]
	GI #4	1700100..1712099	Hypothetical protein SAV1853, rev[1700906..1701056]
			Hypothetical protein SAV1854, rev[1701080..1701680]
			tRNA (cytidine(34)-2'-O)-methyltransferase (EC 2.1.1.207), rev[1701808..1702261]
			Epoxyqueuosine reductase (EC 1.17.99.6) QueG, rev[1702265..1703393]
			Glutamine ABC transporter, ATP-binding protein GlnQ, rev[1703544..1704273]
			ABC transporter, substrate-binding protein (cluster 3, basic aa/glutamine/opines) / ABC transporter, permease protein (cluster 3, basic aa/glutamine/opines), rev[1704259..1705450]
			Membrane protein, rev[1706005..1707067]
	GI #5	1800000..1806099	Phage protein, rev[1800904..1801876]
			Phage tail length tape-measure protein T, rev[1802033..1802579]
			Phage protein, rev[1802592..1802811]
			Hypothetical protein, SAV0881 homolog [SA bacteriophages 11, Mu50B], rev[1802865..1803015]
			Hypothetical protein, SAV0877 homolog [SA bacteriophages 11, Mu50B], rev[1803011..1803218]
			Dimeric dUTPase (EC 3.6.1.23), rev[1803254..1803653]
			Hypothetical protein, PVL orf52 homolog [SA bacteriophages 11, Mu50B], rev[1803775..1804027]
			Hypothetical protein, Lmo2313 homolog [Bacteriophage A118], rev[1804297..1804747]
			Hypothetical protein, PVL orf51 homolog [SA bacteriophages 11, Mu50B], rev[1804847..1805090]
			Hypothetical protein, PVL orf50 homolog [SA bacteriophages 11, Mu50B], rev[1805093..1805462]
			Phage Holliday junction resolvase, rev[1805474..1805879]

	GI #6	1952100..1964099	Mannitol-1-phosphate 5-dehydrogenase (EC 1.1.1.17), dir[1952400..1953507]<br>FmtB (Mrp) protein involved in methicillin resistance and cell wall biosynthesis, rev[1953825..1954038]<br>FmtB (Mrp) protein involved in methicillin resistance and cell wall biosynthesis, rev[1954051..1954423]<br>FmtB (Mrp) protein involved in methicillin resistance and cell wall biosynthesis, rev[1954554..1954830]<br>FmtB (Mrp) protein involved in methicillin resistance and cell wall biosynthesis, rev[1954978..1955842]<br>FmtB (Mrp) protein involved in methicillin resistance and cell wall biosynthesis, rev[1955919..1956084]<br>Phosphoglucosamine mutase (EC 5.4.2.10) @ FemD, factor involved in methicillin resistance, rev[1956360..1957716]<br>Uncharacterized secreted protein associated with spyDAC, rev[1957742..1958675]<br>Diadenylate cyclase spyDAC<br>Bacterial checkpoint controller DisA with nucleotide-binding domain, rev[1958676..1959486]<br>Arginase (EC 3.5.3.1), rev[1959674..1960583]
***S. aureus* OD028**			
	GI #2	492000..502099	Deoxyguanosine kinase (EC 2.7.1.113), rev[492188..492701]<br>tRNA-specific adenosine-34 deaminase (EC 3.5.4.33), dir[492767..493238]<br>Hydrolase, HAD superfamily, dir[493384..494224]<br>Predicted flavoprotein, dir[494244..494811]<br>Antiadhesin Pls, binding to squamous nasal epithelial cells, dir[495090..495582]<br>Adhesin of unknown specificity SdrD, dir[495714..496857]<br>Adhesin of unknown specificity SdrD, dir[496831..498838]<br>Adhesin of unknown specificity SdrE, similar to bone sialoprotein-binding protein Bbp, dir[498942..499428]
	GI #4	1694100..1706099	Hypothetical protein SAV1852, rev[1694484..1694880]<br>Hypothetical protein SAV1853, rev[1695402..1695552]<br>Hypothetical protein SAV1854, rev[1695576..1696176]<br>tRNA (cytidine(34)-2'-O)-methyltransferase (EC 2.1.1.207), rev[1696299..1696764]<br>Epoxyqueuosine reductase (EC 1.17.99.6) QueG, rev[1696768..1697896]<br>Glutamine ABC transporter, ATP-binding protein GlnQ, rev[1698046..1698775]<br>ABC transporter, substrate-binding protein (cluster 3, basic aa/glutamine/opines) / ABC transporter, permease protein (cluster 3, basic aa/glutamine/opines), rev[1698761..1699856]<br>Membrane protein, rev[1700444..1701506]
	GI #5	1788100..1798099	Phage lysin, N-acetylmuramoyl-L-alanine amidase (EC 3.5.1.28), rev[1788630..1788927]<br>Staphylokinase, rev[1789617..1790109]<br>Phage lysin, N-acetylmuramoyl-L-alanine amidase (EC 3.5.1.28), rev[1790299..1791055]<br>Phage holin, rev[1791066..1791321]<br>Phage protein, rev[1791532..1791667]<br>Phage protein, rev[1791824..1791935]

		Structural protein, phage associated, rev[1792054..1794793]
		Phage protein, rev[1794808..1795780]
		Phage tail length tape-measure protein T, rev[1795956..1796517]
		Phage tail length tape-measure protein T, rev[1796510..1796714]
		Phage protein, rev[1796740..1796941]
		Hypothetical protein, SAV0881 homolog [SA bacteriophages 11, Mu50B], rev[1796995..1797145]
		Hypothetical protein, SAV0877 homolog [SA bacteriophages 11, Mu50B], rev[1797141..1797348]
		Dimeric dUTPase (EC 3.6.1.23), rev[1797384..1797921]
GI #6	1946100..1958099	FmtB (Mrp) protein involved in methicillin resistance and cell wall biosynthesis, rev[1946879..1949648]
		Phosphoglucosamine mutase (EC 5.4.2.10) @ FemD, factor involved in methicillin resistance, rev[1949924..1951280]
		Uncharacterized secreted protein associated with spyDAC, rev[1951306..1952239]
		Diadenylate cyclase spyDAC
		Bacterial checkpoint controller DisA with nucleotide-binding domain, rev[1952240..1953050]
		Arginase (EC 3.5.3.1), rev[1953238..1954147]
